# Microwave-Assisted Extraction of Multiple Trace Levels of Intermediate Metabolites for Camptothecin Biosynthesis in *Camptotheca acuminata* and Their Simultaneous Determination by HPLC-LTQ-Orbitrap-MS/MS and HPLC-TSQ-MS

**DOI:** 10.3390/molecules24040815

**Published:** 2019-02-25

**Authors:** Zhaoxia Jin, Ruyi Wan, Ruxue Yan, Yingying Su, Honglan Huang, Lihan Zi, Fang Yu

**Affiliations:** 1 School of Biological Engineering, Dalian Polytechnic University, Dalian 116034, China; shengwu5022016@163.com (R.W.); swgc502@163.com (R.Y.); cxsu2016@163.com (Y.S.); 2 College of Basic Medical Sciences, Jilin University, Changchun 130021, China; shengwu501@126.com; 3 School of Life Science and Biotechnology, Dalian University of Technology, Dalian 116024, China; lihanzi@dlut.edu.cn

**Keywords:** microwave assisted extraction, Taguchi experimental design, metabolites, camptothecin, HPLC-LTQ-MS/MS

## Abstract

Camptothecin (CPT) has strong antitumor activity and is used as an anticancer therapeutic agent. To better understand and decipher the pathway of CPT biosynthesis in *Camptotheca acuminata*, the main purpose here was focused on creating an effective extraction strategy for a rich intermediate metabolite profile. In the present study, a 70% aqueous acetonitrile was verified as an optimal extraction solvent for microwave-assisted extraction (MAE) of metabolites by spiking experiments. Based on multi-objective optimization, the best extraction conditions of a solid-liquid ratio of 1:20, microwave power of 230 W, and a time of 4 min were achieved using a full factorial 3^4^ experimental design. Crude extracts obtained from the shoot apex of *C. acuminata* using MAE have been qualitatively profiled by high-performance liquid chromatography coupled with linear ion trap quadrupole-orbitrap mass spectrometry (HPLC-LTQ-Orbitrap-MS/MS) and a HPLC triple quadrupole-MS (HPLC-TSQ-MS) analysis was conducted for their metabolite content in different tissues. CPT, and ten related metabolites and their isomers, including tryptamine, loganic acid, secologanic acid, strictosidinic acid, strictosamide, strictosamide epoxide, strictosamide diol, strictosamide ketolactam, pumiloside, and deoxypumiloside, were detected and tentatively identified. Scanning electron microscopy (SEM) imaging of the shoot apex demonstrated that severe cell disruption was evident after intensified extraction processes. The study showed the difference of metabolite profiles and the enhancement of metabolite content after microwave-pretreated techniques, and the established MAE procedure is an effective methodology to preserve valuable metabolite compounds for analysis.

## 1. Introduction

Camptothecin (CPT)—a monoterpenoid indole alkaloid (MIA)—was first isolated from the woody medicinal plant *Camptotheca acuminata* Decne [1]. As one of the most promising natural antitumor and antiviral drugs, CPT and its derivatives are used in clinical treatment and are paid more close attention [2,3]. However, because the low yield of CPT is observed among various plant tissues, this leads to its expensive price and inadequate supply in the medical market [4]. To ensure a sufficient supply and gain access to the compounds, promoting steady accumulation of CPT in plants is recognized as the most imperative and feasible strategy, which relies on in-depth investigation and comprehension of the CPT biosynthetic process [5]. Like many other MIAs, CPT is derived from the common central alkaloid precursor strictosidine, which is synthesized by coupling indole-containing tryptamine and the monoterpenoid glycoside secologanin under the catalysis of strictosidine synthase [6,7]. In the upstream MIA pathway, tryptamine is produced through the shikimate pathway by decarboxylation of tryptophan, while secologanin is from the mevalonate (MVA) pathway or 2-methyl-(d)-erythritol-4-phosphate (MEP) pathway [8,9,10,11]. However, at present, the downstream pathway of CPT biosynthesis, namely the pathway following strictosidine (isovincoside), which is an important and specific metabolic pathway, is yet unclear [5,8,9]. Therefore, the extraction and detection of intermediate metabolites in the downstream pathway are urgently needed.

In plants, the biosynthesis of CPT is a complicated and multi-step process in which may generate multiple complex secondary metabolites. In order to analyze all of the intermediates from *C. acuminata* and hence illuminate the biosynthesis pathway, both an efficient metabolite extraction protocol and identification of chemical constituents are fundamental and critical, and have important consequences for the accuracy of phytochemical studies. Conventionally, alkaloid compounds from botanical materials are extracted using traditional methods including grinding extraction, stirring or rotation extraction, ultrasonic extraction, or heat-reflux extraction and maceration extraction at room temperature [12,13,14,15]. However, these extraction procedures have some drawbacks, such as longer extraction times but low extraction efficiencies, being cumbersome, and usually requiring large amounts of solvents [16]. Meanwhile, CPT and almost all intermediate compounds were present at trace levels in plant tissues, and their solubility in organic solvents was poor and different; all these deficiencies may limit the perception and the percentage extraction of pathway metabolites from plant materials. Recently, research on microwave-assisted process intensification and kinetic modelling about extraction of CPT from *Nothapodytes nimmoniana* was reported by Patil et al. [17]. In addition, the ionic-liquids-based microwave-assisted extraction (MAE) method for CPT and its hydroxylated product from samara of *C. acuminata* were developed by Wang et al. [16]. Therefore, a process optimization and intensification to preserve possible intermediate compounds and improvement of extraction efficiency can be achieved with the application of microwave-assistant technologies. On the other hand, in plant secondary metabolites profiling studies, a rapid, convenient, sensitive, and specific detection method, for example, research on the mass spectrometry fragmentation of pathway intermediates in *C. acuminata* was seldom reported [18]. As an important modern analytical tool, HPLC-MS/MS assay with the multiple reaction monitoring modes is more suitable for the identification of the compounds in complex matrixes at relatively low concentrations due to its excellent sensitivity and accuracy. Normally, for qualitative identification of compounds, a high resolution quadrupole-orbitrap MS is the preferred method over low resolution TSQ-MS since it gives a more accurate mass. Meanwhile, TSQ-MS is more typically used as a confirmation approach because of the better sensitivity performance in quantification achieved. However, research has not been systematically carried out on analysis of CPT and intermediates. Thus, herein the reliable high-performance liquid chromatography coupled with linear ion trap quadrupole-orbitrap mass spectrometry (HPLC-LTQ-Orbitrap-MS/MS) mass spectrometer was employed for quick detection and simultaneous identification of various compounds of metabolic pathways.

In the present study, microwave-assisted extraction (MAE) in combination with HPLC- electrospray ionization (ESI)-LTQ-Orbitrap-MS/MS for qualitative identification, and quantitative determination of biosynthesis intermediates with triple quadrupole (TSQ) Quantum tandem HPLC-MS in the extracts of *C. acuminata* was conducted. The extraction parameters, including extraction solvents, solid liquid ratio, microwave irradiation power, and extraction time, were optimized by orthogonal array design. Moreover, one-step quick identification of the untargeted compounds was analyzed, and the simultaneous qualitative determination of seventeen iridoids and secoiridoids in the shoot apex tissues of *C. acuminata* was developed. To the best of our knowledge, this is the first work that reports the profiling of iridoids and secoiridoids in *C. acuminata* by MAE-HPLC-LTQ-Orbitrap-MS.

## 2. Materials and Methods

### 2.1. Chemicals and Reagents

HPLC-grade methanol, acetonitrile was purchased from Oceanpak Alexative Chemical Co., Ltd. (Sweden). Standards of CPT, tryptamine, loganic acid, strictosamide, and telmisartan were purchased from Aladdin Industrial Corporation (Shanghai, China).

### 2.2. Plant Materials

Seed germination and plant growth were conducted as described by Jin et al. [8]. Briefly, seeds were germinated on wet filter paper in a growth chamber with a 12 h photoperiod at 25 °C until cotyledons appeared. The *C. acuminata* seedlings were then transplanted to pots with autoclaved soil on shelves in the greenhouse with a 16 h photoperiod under 25 °C. The plants were grown for one month and the plant tissues were collected. A certain amount of plant tissues (root, shoot apex, young leaf, mature leaf) were dried in an oven at 55 °C for 12 h to constant weight, and then ground into a powder.

### 2.3. Sample Preparation and MAE Procedure

An ETHOS microwave extracting device (Milestone, Italy) was used for the MAE of CPT and intermediates according to Tan et al. [19]. For each run, one gram of plant powder sample was mixed with 30 mL of acetonitrile/water (7/3, *v*/*v*) and was placed into a 100 mL vessel of a microwave reactor. For extraction solvent selection, standards mixture including loganic acid, strictosamide, and CPT was spiked in the sample to achieve a certain target concentration. The extraction procedure was carried out in triplicate under different extraction conditions, and the extracts were combined after centrifugation. The extract was concentrated and dried by rotary evaporation under reduced pressure, and the solid residue was dissolved in 5.0 mL of methanol for their subsequent qualitative analysis.

The quantification of the spiked samples was based on peak areas and was performed using the internal standard calibration approach. The extraction recovery (ER, *n* = 3) for MAE was calculated as the following equation:

ER = [(experimental analytes content in spiked sample − analytes content in unspiked sample)/spiked value] × 100%.

### 2.4. Optimization of the Extraction Parameters Based on the Taguchi Design

Three extraction parameters of solid to liquid ratio, irradiation power, and extraction time, each with three levels comprising nine experiments were designed and tested using the Taguchi orthogonal array method [20]. Each trial was conducted in triplicate and the average analyte concentration and compound number was analyzed to optimize extraction conditions for MAE. The statistical significances and significant factors were determined from the orthogonal experimental data by using either the analysis of range (ANORA) or one-way analysis of variance (ANOVA). Both the F-value and *p*-value were used to evaluate the significance of the process parameters, and a *p* value ≤0.05 was considered as statistically significant.

### 2.5. Determination of Metabolites in C. acuminata by HPLC-LTQ-Orbitrap-MS/MS and HPLC-TSQ-MS

Qualitative determination of metabolites of camptothecin biosynthesis in *C. acuminata* was performed by HPLC-LTQ-Orbitrap-MS/MS. Prior to HPLC/MS analysis, each sample of 475 μL (100 μL extract + 375 μL deionized water) was added with 25 μL of 0.001% formic acid and was then filtered through a 0.22 μm hydrophobic membrane. A mixed standard solution in methanol containing available standards of CPT, loganic acid, and strictosamide in concentrations of 150 µg/mL, respectively, were prepared as reference, and telmisartan was used as an internal standard. The LC-MS^*n*^ detection was performed on an LTQ Orbitrap XL fitted with an ESI source, and operated in positive ion mode, in a mass range of 120 to 1000. A 31 min gradient elution with mobile phases composed of 10 mM aqueous ammonium formate (solvent A, adjusted to pH 2.83 with formic acid)-methanol (solvent B) was used. The gradient elution program is briefly described as follows: 0–15 min, a gradient of 10% up to 95% solvent B; 15–20 min, with a hold at 95% solvent B; 20–21min, returned to starting conditions of 10% solvent B and held until 31 min for re-equilibration. A Hypersil Gold C18 column (150 × 2.1 mm, 5 μm, Thermo) was used and the detection wavelength was 254 nm. The injection volume was 2 μL and the mobile phase flow rate was 0.2 mL/min. Analysis conditions for MS/MS were as follows: source voltage: 3.50 kV; sheath gas (nitrogen): 30 arbitrary units (arb. units); auxiliary gas flow: 10 arb. units; capillary voltage, 39 V; capillary temperature, 350 °C.

Chromatographic quantitative analysis was performed using a HPLC-TSQ-MS system (TSQ Quantum Ultra, Thermo Fisher Scientific) equipped with an electrospray ionization (ESI) interface in positive or negative ion mode. A 31 min gradient elution program was used, and quantitative calculation was carried out by internal standard and the normalization method based on peak areas of analytes. Analysis conditions for MS were as follows: spray voltage: 3.0 kV (positive ion mode); 2.5 kV (negative ion mode); vaporizer temperature: 250 °C; sheath gas pressure: 35 arb. units; auxiliary gas flow: 10 arb. units; ion sweep gas pressure: 0 arb. units; capillary temperature, 350 °C.

### 2.6. Scanning Electron Microscopy (SEM) Analysis

For preparing specimens for the SEM analysis, plant shoot apexes treated under different MAE conditions were oven-dried at 60 °C to constant weight. Three dried powder samples before or after MAE were fixed and observed for surface microstructural characterization using a field emission scanning electron microscope (SEM) (JSM-7800F, JEOL, Tokyo, Japan).

## 3. Results and Discussion

### 3.1. Selection of Extraction Solvents

**Figure 1:**
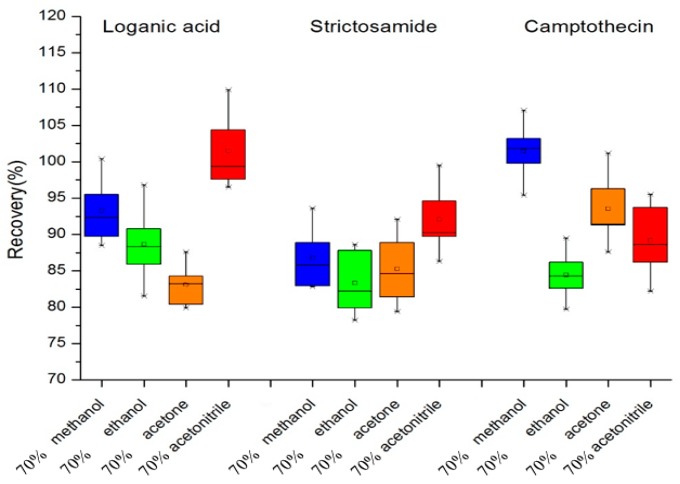
Effects of various extraction solvents on the recoveries of different intermediate compounds by microwave-assisted extraction (MAE). Box-whisker plots show the median (horizontal line inside box), the 25th and 75th percentiles (bottom and top border of box), and the minimum and maximum percentile values of the data (whiskers), respectively.

The primary step in the process of extraction is to select a suitable solvent which exhibits both good solubility for the analytes of interest and good microwave energy absorption performance. The four different solvents (30 mL of methanol, ethanol, acetone, or acetonitrile respective concentration at 70% in water) were tested respectively as extractants mixed with 1 g of plant shoot apex sample. The extractions were performed at a microwave power of 230 W for 4 min. Because all standard compounds were not available, the recoveries of loganic acid, strictosamide, and CPT were used to estimate extraction efficiency of the solvent. Recovery studies were carried out using samples spiked with the three analyte standards at certain concentrations before the analysis. As shown in Figure 1, in the MAE method, when aqueous acetonitrile of 70% was used as the extraction solvent, the recoveries of the three target compounds ranged between 88% and 103%, and acetonitrile had the highest extraction efficiency for two of the key intermediates (loganic acid, strictosamide). The most commonly employed solvent for the extraction of CPT and 10-HCPT in plant samples is methanol [8,13,17]. However, fewer systematic studies on intermediates extraction were reported. Maybe acetonitrile was not the best solvent for all of the given intermediates with discrepant polarity and dielectric constant, however, it should be the best choice for multi-objective optimization, which assessed the efficiency of extraction for multiple analytes in an appropriate level based on a comprehensive perspective. Meanwhile, a certain amount of water in solvent would help in further improving the extraction efficiency by absorption of more microwave energy and facilitate rapid and efficient mass transfer among plant cells by diffusion during extraction [21,22,23]. Thus, aqueous acetonitrile of 70% was chosen as the optimal extracting solvent for MAE.

### 3.2. Optimization of MAE Conditions with Orthogonal Array Testing Influential Factors

The extraction efficiency of MAE can be affected by ambient conditions. Thus, the influences of extraction parameters on the chemical components, contents, and characteristics of crude extracts were subsequently estimated. Based on the preliminary testing results, a three-factor and three-level orthogonal experimental design L_9_ (3^4^) was used for process condition optimization. Solid-liquid ratio (1:10, 1:20, and 1:30 g/mL), microwave power (120 W, 230 W, and 385 W) and extraction time (4 min, 6 min, and 8 min) were the independent variables, as shown in Table 1 and Tables S1–S3, and the combined effects and benefits of these three factors were investigated. To attain efficient extraction of multiple potential intermediate compounds in pathways, multi-criterion estimation, namely two different objective functions including the concentrations of representative compounds and the total number of intermediate compounds, were used here. For this purpose, two key and representative central intermediates of loganic acid and strictosamide were chosen, and each of which was a trade-off between objectives [24,25]. From the results of range analysis of the orthogonal experiment, the order of factor impacts on the extraction efficiency of intermediates content was microwave power > solid-liquid ratio > extraction time, and the effects on the number of intermediates were presented in the same order. Based on further multi-factor analysis of variance, it can be seen that for loganic acid and strictosamide content extraction and occurrence of compounds, the effect of solid-liquid ratio and microwave power was more significant (*p* < 0.05) when using MAE. Thus, a solid-liquid ratio of 1:20 and microwave power of 230 W was selected. Meanwhile, the extraction time of the three levels variable (K_1_, K_2_, K_3_) had marginal impact on two indexes, thus the extraction time 4 min was chosen for timesaving and avoiding solvent evaporation. Therefore, based on the above, the optimal extraction conditions were A2B2C1. Due to process intensification by MAE, the extraction time of MAE was shortened to just 4 min with 9.42 mg/g loganic acid yield and 16.21 mg/g strictosamide yield, while efficiency of the conventional grinding method had much lower yield even after MAE pretreatment for 16 h, as shown in Table S4. Thus, MAE is considered a good extraction method for the studied plant intermediate metabolites.

### 3.3. SEM Analysis

**Figure 2:**
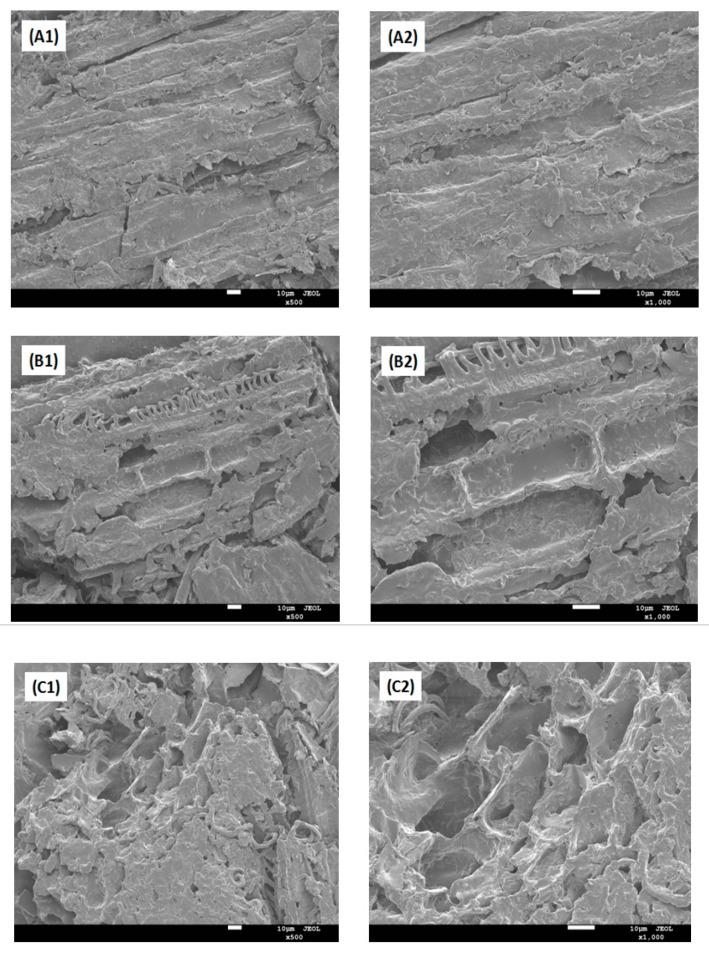
Scanning electron micrographs (SEM) of the samples; (**A1**) control-ground sample × 500; (**A2**) control-ground sample × 1000; (**B1**) microwave-assisted extracted (MAE) sample in min × 500; (**B2**) MAE sample in min × 1000; (**C1**) MAE sample in high × 500; (**C2**) MAE sample in high × 1000.

Microstructural changes in the surface morphologies of plant materials were observed before and after MAE extraction, as shown in Figure 2A–C. As shown in Figure 2(A1,A2), the structure of the plant shoot apex ground with liquid nitrogen but without MAE treatment was kept basically intact and showed only few and slight visual surface impairments. While compared to the conventional extraction method, the MAE process at a moderate operating power condition increased the disruption degree of native tissue structure, and inevitably, plants displayed apparent cell breakage and a few discrete holes on the tissue surface, as shown in Figure 2(B1,B2). Figure 2(C1,C2) present the micrographs of the MAE sample treated with high power. The microscopic observations indicated that microwave irradiation led to a quick rise of temperature and an increase of intracellular pressure, and then the cytoderm opened the loop obviously, the texture crumbled and shrunk, there was accelerated cell cracking, and it caused great damage to surface structure. It has been reported by Patil et al. that there were similar appearance features for the MAE of CPT from the *Nothapodytes nimmoniana* plant [17]. This destruction may increase the percentage extraction and extraction efficiency in a given time. Because of the strong penetrating power ability the microwave has, it may make both the inside and outside parts of the sample be evenly and quickly heated at the same time. Moreover, during the MAE process at high temperature, solvent viscosity decreases and diffusion through the cell membrane increases. Meanwhile, the rupture including the presence of holes or cracks in plant stems should promote intracellular substances exudation into the extracellular matrix. Thus, considering all these factors together, MAE generally works better and provides a high efficiency of extraction than conventional methods [26,27].

### 3.4. Identification of CPT Biosynthetic Intermediates in C. acuminata

To assess the extraction efficiency of MAE and to reveal metabolic pathways in plants, the extracts obtained by MAE under the optimal conditions were analyzed and the identification of main intermediate compounds in CPT biosynthesis was conducted. The ultra-high-performance liquid chromatography coupled with mass spectrometry (UHPLC-MS) and UHPLC-quadrupole-time-of-flight-MS/MS (UHPLC-QTOF-MS/MS) methods have been previously described by Sadre et al. [18]. In our paper, a non-target analytical strategy consisting of the combined use of HPLC-MS and HPLC-MS/MS was used, both with triple-quadrupole tandem mass spectrometry and HPLC-ESI-LTQ-Orbitrap-MS/MS assay. Accurate mass measurements of the positive precursor and product ions masses were simultaneously performed, as shown in Figure S2. Seventeen putative metabolites belonging to iridoids of the CPT pathway were simultaneously annotated and identified based on their retention times, MS and MS/MS data compared with standards, if available, and with those previously published common standards, as shown in Table 2 and Figure S1A1–4 [28,29]. According to the backbone structure of compounds, among the 17 compounds identified, as shown in Figure S1B, 1 tryptamine, 2 iridoids or secoiridoids (loganic acid, secologanic acid), 2 compounds belonging to MIA strictosidinic acid, 3 compounds belonging to the vincosamide group (strictosamide, strictosamide epoxide, strictosamide diol), 8 compounds belonging to the pumiloside group (strictosamide ketolactam, pumiloside, deoxypumiloside), and 1 MIA camptothecin were included [12]. Moreover, the HPLC profiles of extracts showed that many intermediate isomers were produced in the CPT pathway, e.g., two isomers of strictosidinic acid, three isomers of strictosamide ketolactam, two isomers of pumiloside, as well as two isomers of deoxypumiloside were structurally differentiated.

Compared with a 52 min UHPLC-Q-TOF-MS/MS gradient approach reported formerly, a rapid detection and identification and more sensitive and cheaper method for elucidation of the metabolic profile was achieved within 20 min using HPLC coupled to high-resolution mass spectrometry (HPLC-ESI-HRMS) [18,30]. In this study, a hybrid LTQ-Orbitrap high-resolution MS platform was used, and the experimental data and calculated values of detected compounds were found within a mass tolerance of 0.8–9.9 ppm. The molecular structure of each compound was proven by its specific fragmentation patterns and structural information. Some recent studies have indicated that two alternative seco-iridoid pathways are proposed to be participated in plant MIA biosynthesis, either through multiple isomeric intermediates of the methyl ester of strictosidine or the carboxylic acid strictosidinic acid [31]. Our results were coincident with the conclusion that strictosidinic acid was the key MIA precursor and the central intermediate in CPT biosynthesis in *C. acuminata*. However, it was found that the metabolite diversity and the number of isomers of most pathway intermediates identified here were quantitatively different from those of previous investigations, and more trace post-strictosidinic acid intermediates and isomers were measured in our study. For example, strictosidinic acid, strictosamide epoxide, and strictosamide ketolactam was reported to have two isomers, respectively, by Chen et al., while no isomeric forms were observed for strictosamide, pumiloside, and deoxypumiloside [12]. Therefore, the main results of LTQ-MS/MS were the changes in the compounds’ profile after microwave-assisted pretreatments and extraction. The different results of isomers compared to the literature were mainly due to the different detection methods, which needed further verification and research.

### 3.5. Comparative Analysis of Metabolites in Plant Samples

**Figure 3:**
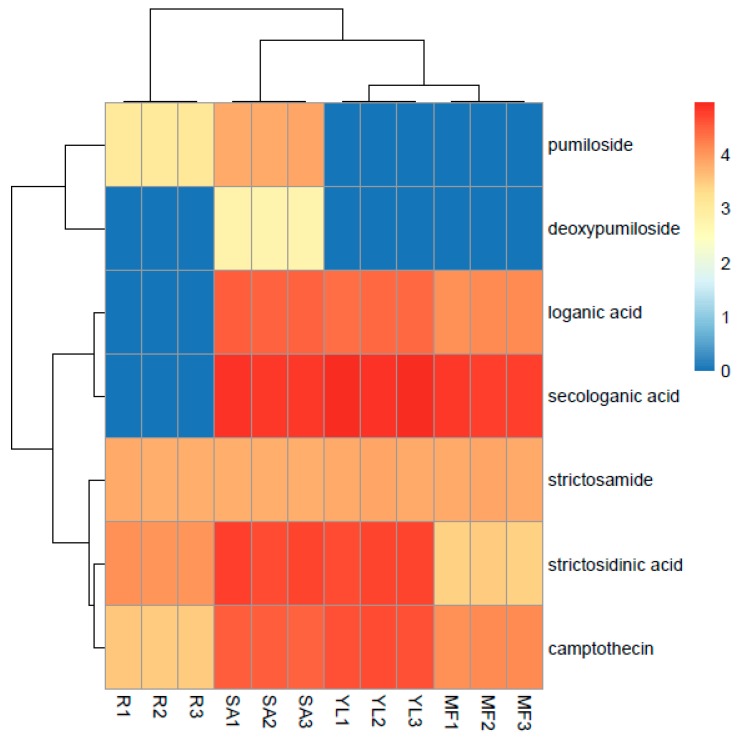
Comparative analysis of intermediate metabolite content in different plant parts after MAE by heat map using raw peak response of HPLC-TSQ-MS. R1–R3: root 1–3; SA1–SA3: shoot apex 1–3; YL1–YL3: young leaf 1–3; ML1–3: mature leaf 1–3.

In order to prove the applicability of the developed extract method, MAE was applied for practical samples analysis. Four different plant tissues, namely root, shoot apex (SA), young leaf (YL), and mature leaf (ML) of *C. acuminata* was used for metabolite analysis in their extracts. The relative levels of the intermediate metabolites in each tissue extract were quantified by HPLC-TSQ-MS, as shown in Figure S3. The resulting heat map is shown in Figure 3, which was generated by raw data of metabolite profiling. Colors in the heat map highlight the visual difference of metabolites and their relative levels in each tissue. It was found that the shoot apex from *C. acuminata* plantlets under greenhouse conditions accumulated relatively most abundant metabolite profiling at higher levels than other plant tissue samples, followed by young leaf. The similarity between chromatograms of SA and YL were 71.4%, whereas the similarity values of SA and the other tissues (root and ML) were 57.1%, which suggested that there existed some certain differences in intermediate metabolite composition and metabolite content between different plant tissues matrixes. In our study, pumiloside and deoxypumiloside were not detected in ML and root tissue samples, respectively, which were contrary to the results obtained by Sadre et al. [18]. It was reported that concentrations of secondary metabolite CPT and intermediates vary among different species of *C. acuminata* and are related to tissue ages and seasons [32,33,34]. Therefore, we can deduce that the species differences may lead to the detection differences of pumiloside and deoxypumiloside in contrast to previous studies.

## 4. Conclusions

In current work we described the development of a feasible MAE method combining HPLC-MS/MS detection to extract and analyze metabolites in *C. acuminata*. Taguchi orthogonal array design was performed to evaluate and optimize multiple factors in MAE. Optimum MAE conditions of solid-liquid ratio, microwave power, and extraction time were established for the first time, which resulted in enhanced metabolite content and composition with reduced extraction time compared to samples without MAE. HPLC-LTQ-Orbitrap-MS/MS analysis further confirmed that the MAE process was efficient and had positive effects on the metabolite profile. The advantages of the developed method included fast and uniform heat transfer, lower consumption of extraction solvent, and simplicity and rapidity of operation for extraction and detection. The applicability of the MAE method to actual plant samples by HPLC-TSQ-MS demonstrated that good performance was attained in extraction efficiency, recovery, and reproducibility of various metabolites in camptothecin biosynthesis.

## Figures and Tables

**Table 1:** The Taguchi L_9_ (3^4^) orthogonal array in terms of multiple factors at three levels and their effects on extraction efficiency of intermediates.

	Run	A Solid-Liquid Ratio (g/mL)	B Microwave Power (W)	C Extraction Time (min)	Intermediates Content		Total Number of Intermediate Compounds
					Loganic Acid (mg/g DW)	Strictosamide (mg/g DW)	
	1	1:10	120	4	2.68	2.94	5
	2	1:10	230	6	7.14	14.06	7
	3	1:10	385	8	5.63	8.23	6
	4	1:20	120	6	1.76	4.63	7
	5	1:20	230	8	9.42	16.21	8
	6	1:20	385	4	4.78	6.98	7
	7	1:30	120	8	0.96	1.38	4
	8	1:30	230	4	6.45	10.72	6
	9	1:30	385	6	4.03	5.67	5
Strictosidinic acid	K_1_	5.15	1.8	4.64	Factors ordered by significance: Microwave power > Solid liquid ratio > Extraction time Optimal parameter: B2 A2C2		
	K_2_	5.32	7.67	4.31			
	K_3_	3.81	4.81	5.34			
	R	1.51	5.87	1.03			
Strictosamide	K_1_	8.41	2.98	6.88	Factors ordered by significance: Microwave power > Solid liquid ratio > Extraction time Optimal parameter: B2 A2C2		
	K_2_	9.27	13.66	8.12			
	K_3_	5.92	6.96	8.61			
	R	3.35	10.68	1.24			
Occurrence of compounds	K1	6	5.33	6	Factors ordered by significance: Microwave power > Solid liquid ratio > Extraction time Optimal parameter: B2 A2C2		
	K2	7.33	7	6.33			
	K3	5	6	6			
	R	1.33	1.67	0.33			

A1: 1:10; A2: 1:20; A3: 1:30; B1: 120 W; B2: 230 W; B3: 385 W; C1: 4 min; C2: 6 min; C3: 8 min.

**Table 2:** Non-targeted analysis of the metabolic profile of intermediate compounds identified from the shoot apex of *C. acuminata* using HPLC-electrospray ionization (ESI) (+)-linear ion trap quadrupole (LTQ)-Orbitrap-MS/MS after MAE.

Peak No.	RT (min)	Theoretical [M + H]^+^ (*m/z*)	Measured [M + H]^+^ (*m/z*)	MS/MS Fragment(s)	Molecular Formula	Compound Identification
1	7.99	394.1708 ^a^ [M + NH_4_]^+^	394.1703 ^a^ [M + NH_4_]^+^	359.1332, 341.1227, 323.1077, **215.0910** ^c^, 197.0807, 179.0701, 151.0747, 137.0581, 109.0654	C_16_H_2__4_O_10_	Loganic acid
2	8.01	533.2130	533.2183	**371.1678**	C_26_H_32_N_2_O_10_	Strictosamide diol
3	8.40	161.1073	161.1070	144.0788	C_10_H_12_N_2_	Tryptamine
4	8.98	397.1105 ^b^ [M + Na]^+^	397.1099 ^b^ [M + Na]^+^	**235.0573**, 217.0468	C_16_H_22_O_10_	Secologanic acid
5	10.16	531.1973	531.1968	**369.1441**, 351.1333, 299.1024 369.1442, 351.1236, 299.1024 281.0915, 176.0540, 158.0594 369.1443, 351.1331, 299.1022 281.0917, 158.0579	C_26_H_30_N_2_O_10_	Strictosamide ketolactam I
6	10.53	517.2181	517.2173	500.1907, **355.1648**, 338.1383, 320.1279, 170.0960 500.1909, 355.1648	C_26_H_32_N_2_O_9_	Strictosidinic acid I
7	10.65	513.1868	513.1861	**351.1335**, 333.1226, 281.0918	C_26_H_28_N_2_O_9_	Pumiloside I
8	10.83	515.2024	515.2016	**353.1493**, 335.1365, 283.1079	C_26_H_30_N_2_O_9_	Strictosamide epoxide
9	11.46	531.1973	531.1968	**369.1442**, 351.1236, 299.1024 281.0915, 176.0540, 158.0594	C_26_H_30_N_2_O_10_	Strictosamide ketolactam II
10	11.76	517.2181	517.2173	500.1909, **355.1648**	C_26_H_32_N_2_O_9_	Strictosidinic acid II
11	11.98	513.1868	513.1861	**351.1335**, 333.1233, 305.1276 281.0921	C_26_H_28_N_2_O_9_	Pumiloside II
12	13.55	349.1183	349.1178	305.1285, 277.1324, 249.1017	C_20_H_16_N_2_O_4_	Camptothecin
13	13.69	513.1868	513.1861	**351.1336**, 333.1232, 315.1125 281.0921	C_26_H_28_N_2_O_9_	Pumiloside III
14	13.79	497.1918	497.1914	**335.1388**, 265.0973, 247.0868 219.0914, 169.0761, 142.0654	C_26_H_28_N_2_O_8_	Deoxypumiloside I
15	14.41	531.1973	531.1968	**369.1443**, 351.1331, 299.1022 281.0917, 158.0579	C_26_H_30_N_2_O_10_	Strictosamide ketolactam III
16	14.49	497.1924	497.1913	**335.1384**, 265.0970, 247.0868	C_26_H_28_N_2_O_8_	Deoxypumiloside II
17	15.80	499.2075	499.2065	**337.1546**, 267.1119	C_26_H_30_N_2_O_8_	Strictosamide

I, II, II stand for isomers; RT: retention time; ^a^: precursor ion [M + NH^4^]^+^; ^b^: precursor ion [M + Na]^+^; ^c^: the MS/MS fragments in bold stand for fragment ions generated after one glucose loss.

## Supplementary Materials

The following are available online.
