# Supplementary material for: Microwave-Assisted Extraction of Multiple Trace Levels of Intermediate Metabolites for Camptothecin Biosynthesis in Camptotheca acuminata and Their Simultaneous Determination by HPLC-LTQ-Orbitrap-MS/MS and HPLC-TSQ-MS
Source: Molecules. 2019 Feb 25;24(4):815. doi: 10.3390/molecules24040815 (PMC6413206; doi:10.3390/molecules24040815)
Supplement: Supplementary file 1 [file molecules-24-00815-s001.pdf]

Microwave assisted extraction of multiple trace levels of intermediate metabolites for camptothecin biosynthesis in *Camptotheca acuminate* and their simultaneous determination by HPLC-linear ion trap-orbitrap-MS/MS

Zhaoxia Jin [1,*], Ruyi Wan [1], Ruxue Yan [1], Yingying Su [1], Honglan Huang [2], Lihan Zi [3] and Fang Yu [1,*]

1. School of Biological Engineering, Dalian Polytechnic University, Dalian 116034, China; 2. College of Basic Medical Sciences, Jilin University, Changchun, 130021, China; 3. School of Life Science and Biotechnology, Dalian University of Technology, Dalian, Liaoning, 116024, China.

MAE-based multiple metabolites analysis by HPLC-LTQ-Orbitrap-MS/MS

[*] Corresponding author.

E-mail: fyu0506@gmail.com; jinzx2018@163.com

Tel: +86-411-86323646; Fax: +86-411-86323646.

Supporting Information

Table S1 Analysis of variance (ANOVA) for the MAE of strictosidinic acid

Factor	Sum of Squares	Degree of Freedom	Mean of Squares	F-value	p	Significance
Solid liquid ratio	4.086	2.00	2.043	1292.81	0.02	*
Microwave Power	51.698	2.00	25.849	2229.31	0.015	*
Extraction Time	1.651	2.00	0.825	862.81	0.02	*
Error	1.859	2.00	0.929			
Total	59.293	8.00				

Table S2 Analysis of variance (ANOVA) for the MAE of strictosamide

Factor	Sum of Squares	Degree of Freedom	Mean of Squares	F-value	p	Significance
Solid liquid ratio	18.151	2.00	9.076	18.901	0.05	*
Microwave Power	174.811	2.00	87.405	182.027	0.005	*
Extraction Time	4.756	2.00	2.378	4.952	0.168	
Error	0.960	2.00	0.480			
Total	198.678	8.00				

Table S3 Analysis of variance (ANOVA) for the MAE of the occurrence of compounds

Factor	Sum of Squares	Degree of Freedom	Mean of Squares	F-value	p	Significance
Solid liquid ratio	8.222	2.00	4.11	37.00	0.03	*
Microwave Power	4.222	2.00	2.11	19.00	0.05	*
Extraction Time	0.222	2.00	0.11	1.00	0.50	
Error	0.222	2.00	1.11E-01			
Total	12.89	8.00				

Table S4 Comparison of MAE method and other extraction methods under the optimal conditions

Metabolites	Matrix	Extraction method	Extraction time	Extraction solvent	Yield/ Extraction efficiency	References
CAM	Samara	ME	24 h	Ethanol (85%)	242.6 μg/g DW	[16]
CAM	Samara	MAE	8 min	IL	674.5 μg/g DW	[16]
CAM	Samara	UAE	35 min	IL	00%	[13]
CAM analogues	Fruits	SPE	60 min	Methanol	A total of 30 compounds identified	[12]
LA, STR, CAM	Shoot apex	MAE	4 min	Acetonitrile (70%)	9.42, 16.21, 18,08 mg/g DW	Our study

Metabolites: LA: Loganic acid; STR: Strictosamide; CAM: Camptothecin.
Method: ME: Maceration extraction; UAE: Ultrasonic-assisted extraction; SPE: Bilayer solid-phase extraction
Solvent: IL: Ionic liquid-aqueous solution;
Yield: DW: Dry weight;

Fig. S1

A1-A4: The TIC chromatogram of *C. acuminata* extract obtained by MAE method based on raw data from HPLC-LTQ-Orbitrap-MS/MS. Each chromatographic peak of 1-17 in figure A1-A4 is corresponding to the chemical structures of 1-17 in the following figure B.

B: Chemical structures of metabolite compounds identified in shoot apex of *C. acuminate*

Fig. S2 MS spectra data for the identification of putative compounds.

A: Loganic acid; B: Strictosamide diol; C: Tryptamine; D: Secologanic acid;

E, I, O: Strictosamide Ketolactam I, II, III; F, J: Stricyosidinic acid I,II;

G, K, M: Pumiloside I, II, III; H: Strictosamide epoxide; L: Camptothecin;

N, P: Deoxypumiloside I, II; Q: Strictosamide.

Fig. S3 Chromatograms (Fig.S3) for compounds quantitative analysis in plant tissues.

A: Root; B: Shoot apex; C: Young leaf; D: Mature leaf.

Fig.S1

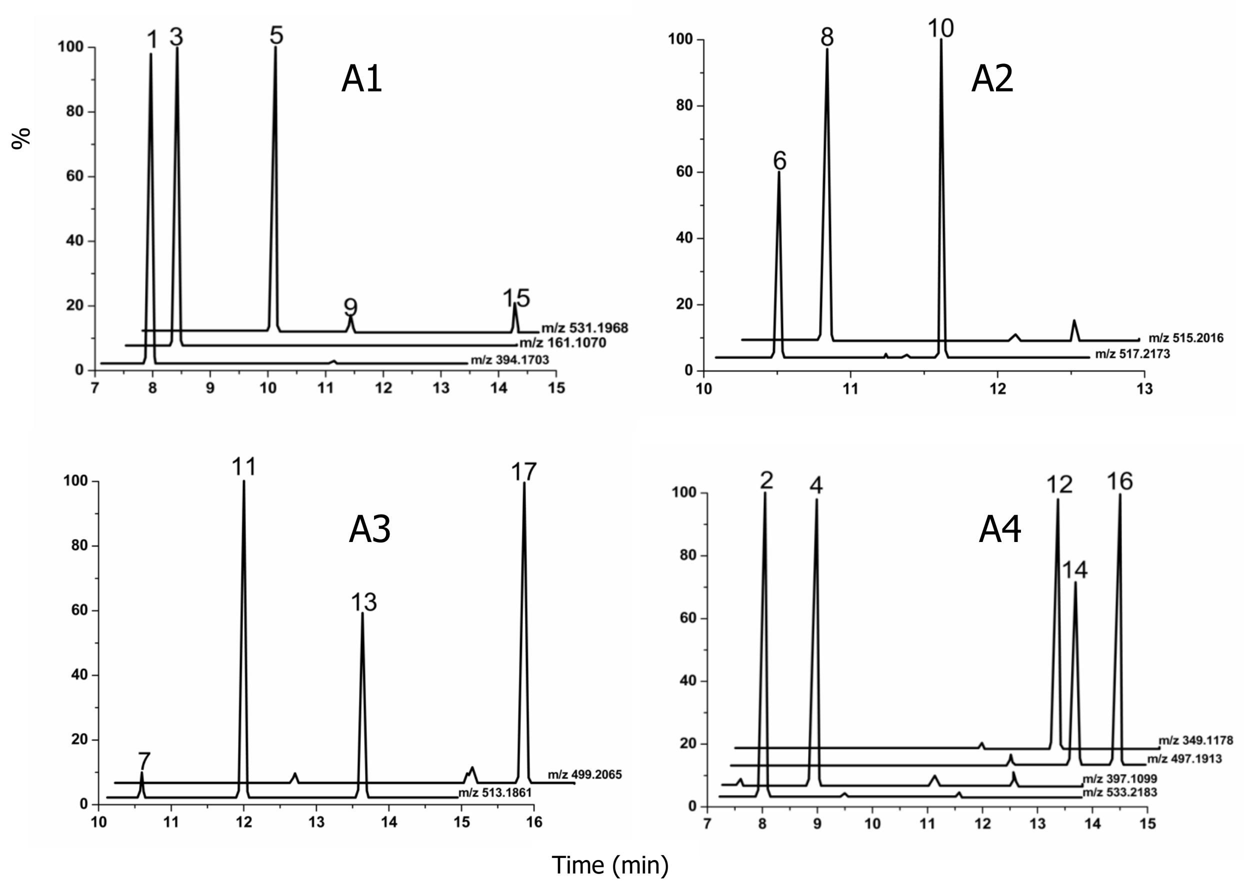

B

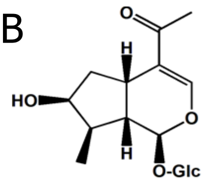

Peak1: Loganic acid

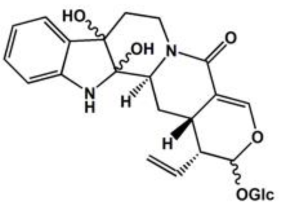

Peak2: Strictosamide diol

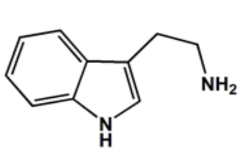

Peak3: Tryptamine

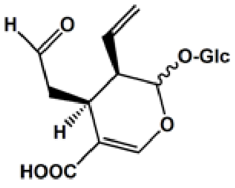

Peak4: Secologanic acid

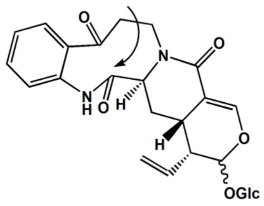

Peak5, 9, 15: Strictosamide Ketolactam I II III

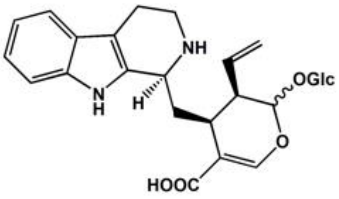

Peak6, 10: Stricyosidinic acid I II

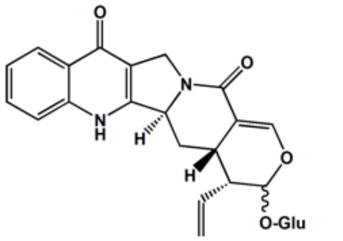

Peak7, 11, 13: Pumiloside I II III

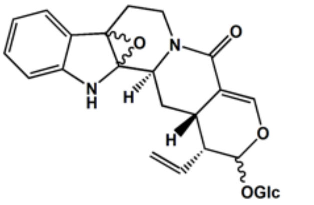

Peak8: Strictosamide epoxide

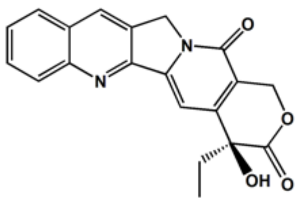

Peak12: Camptothecin

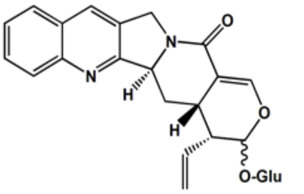

Peak14, 16: Deoxypumiloside I II

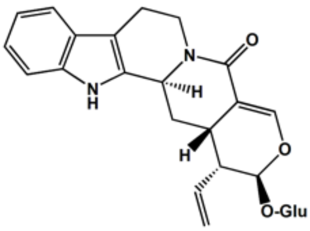

Peak17: Strictosamide

Fig.S2

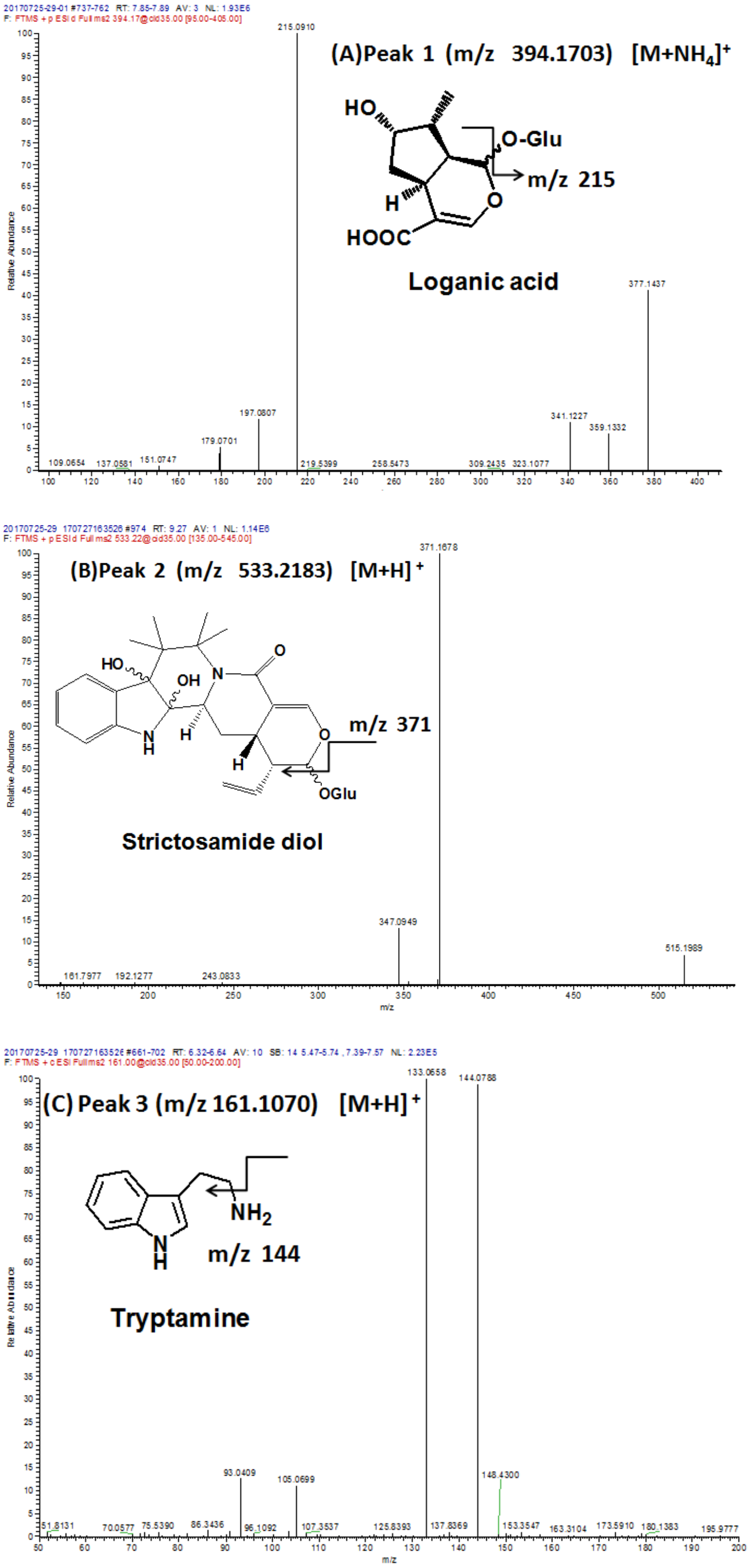
(A)Peak 1 (m/z 394.1703) [M+NH4]+
HO
O-Glu
m/z 215
H
O
HOOC
Loganic acid
215.0910
377.1437
197.0807
341.1227
359.1332
179.0701
Relative Abundance
(B)Peak 2 (m/z 533.2183) [M+H]+
HO
OH
N
O
m/z 371
H
OGlu
Strictosamide diol
371.1678
347.0949
515.1989
m/z
(C) Peak 3 (m/z 161.1070) [M+H]+
NH2
N
H
m/z 144
Tryptamine
133.0658
144.0788
93.0409
105.0699
148.4300

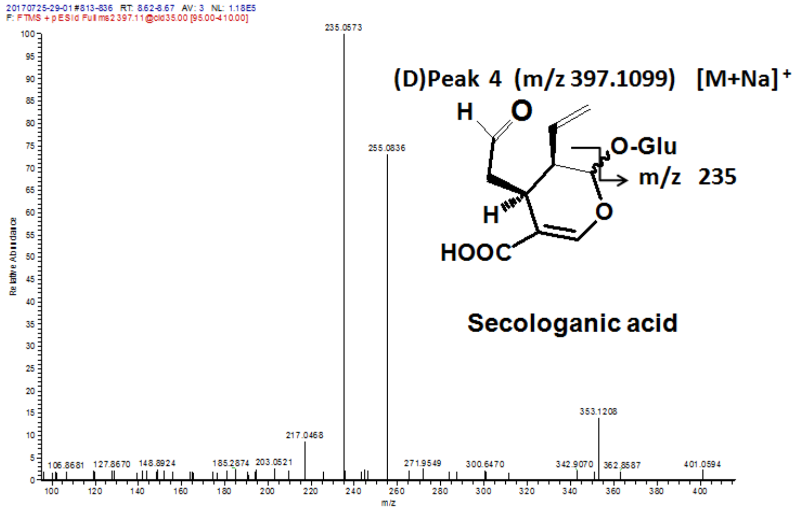
(D)Peak 4 (m/z 397.1099) [M+Na]+
H
O
O-Glu
m/z 235
H
O
HOOC
Secologanic acid
235.0573
255.0836
217.0468
353.1208
Relative Abundance
m/z

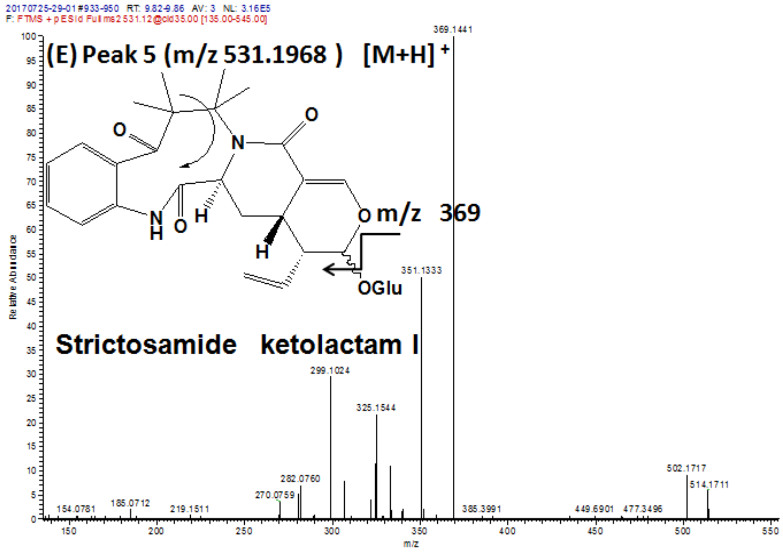
(E) Peak 5 (m/z 531.1968) [M+H]+
O
N
O
NH
O
H
H
O
m/z 369
OGlu
Strictosamide ketolactam I
369.1441
351.1333
299.1024
325.1544
282.0760
270.0759
502.1717
514.1711
Relative Abundance
m/z

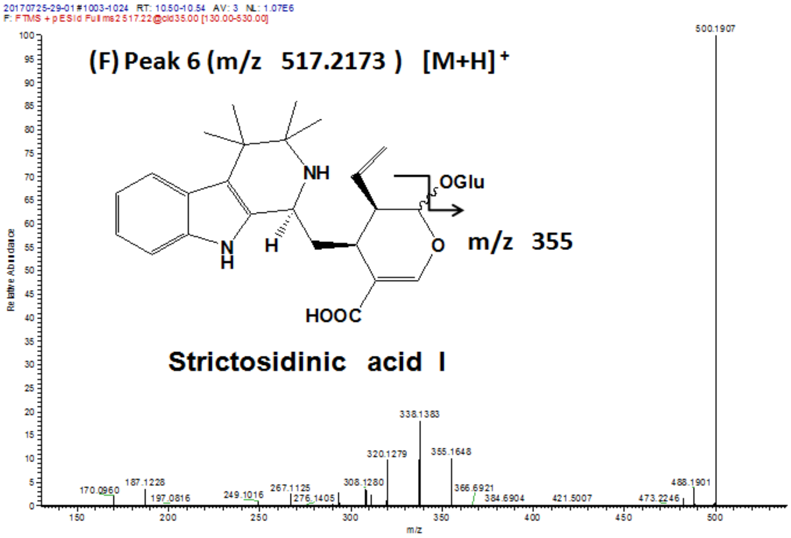
(F) Peak 6 (m/z 517.2173) [M+H]+
NH
OGlu
NH
H
O
m/z 355
HOOC
Strictosidinic acid I
500.1907
338.1383
355.1648
320.1279
308.1280
488.1901
Relative Abundance
m/z

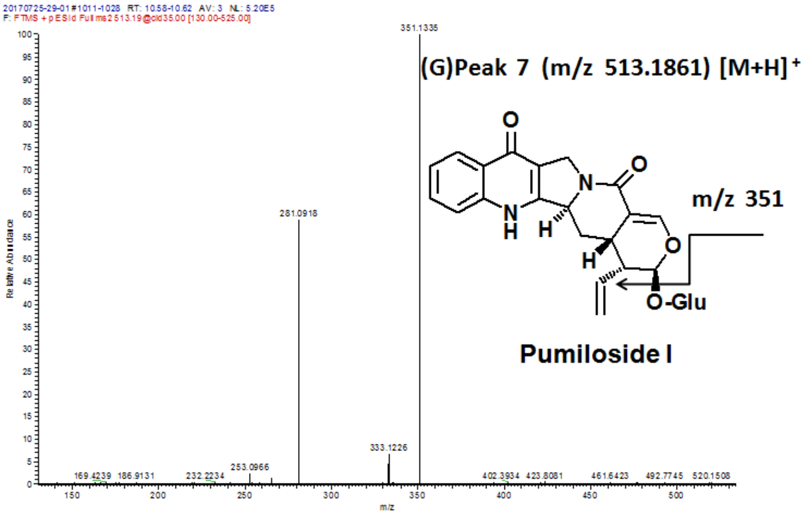

20170725-29-01 #1011-1028 RT: 10.58-10.62 AV: 3 NL: 5.20E5
F: FTMS + p ESI d Full ms2 513.19@cid35.00 [130.00-525.00]
(G)Peak 7 (m/z 513.1861) [M+H]+
m/z 351
O-Glu
Pumiloside I
351.1335
281.0918
333.1226
253.0966
Relative Abundance
m/z

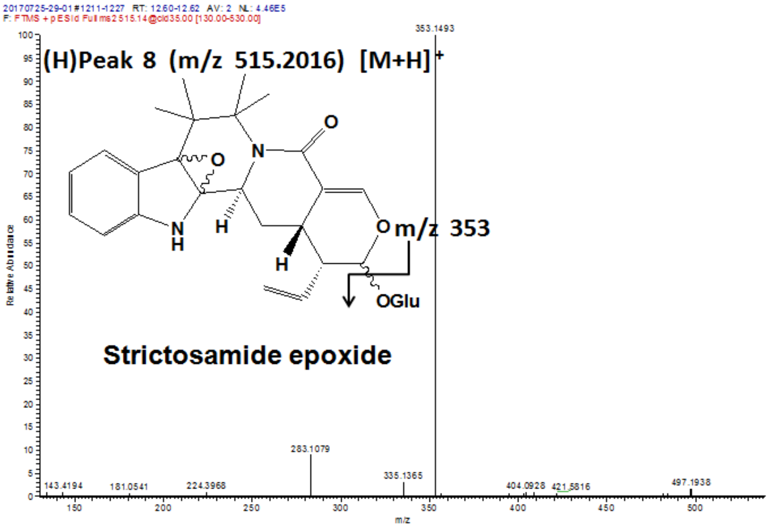

20170725-29-01 #1211-1227 RT: 12.60-12.62 AV: 2 NL: 4.46E5
F: FTMS + p ESI d Full ms2 515.14@cid35.00 [130.00-530.00]
(H)Peak 8 (m/z 515.2016) [M+H]+
m/z 353
OGlu
Strictosamide epoxide
353.1493
283.1079
335.1365
497.1938
Relative Abundance
m/z

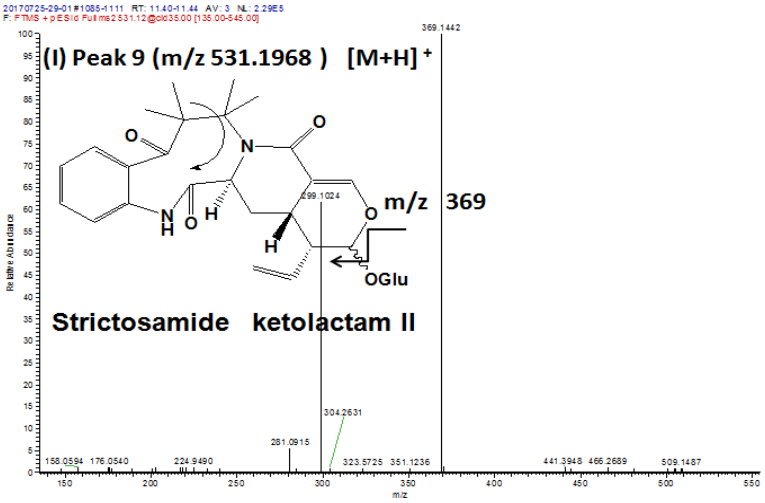

20170725-29-01 #1085-1111 RT: 11.40-11.44 AV: 3 NL: 2.29E5
F: FTMS + p ESI d Full ms2 531.12@cid35.00 [135.00-545.00]
(I) Peak 9 (m/z 531.1968) [M+H]+
m/z 369
OGlu
Strictosamide ketolactam II
369.1442
304.2631
281.0915
Relative Abundance
m/z

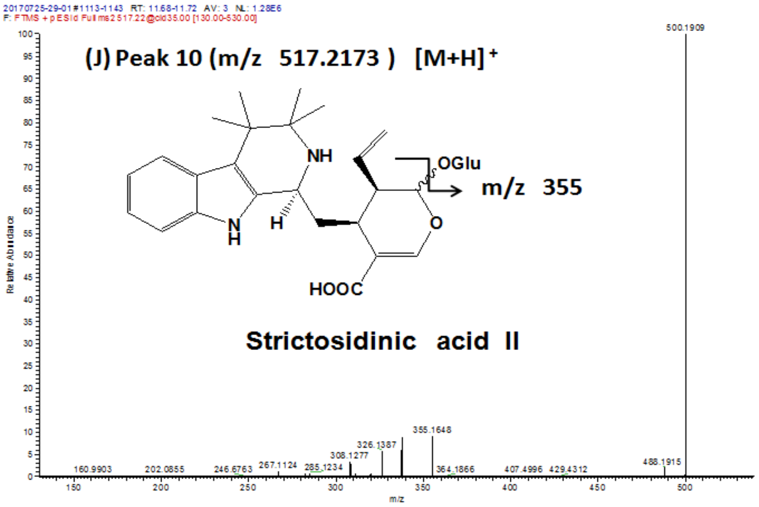
(J) Peak 10 (m/z 517.2173) [M+H]+
NH
OGlu
m/z 355
N
H
H
O
HOOC
Strictosidinic acid II
500.1909
355.1648
326.1387
308.1277
488.1915
Relative Abundance
m/z

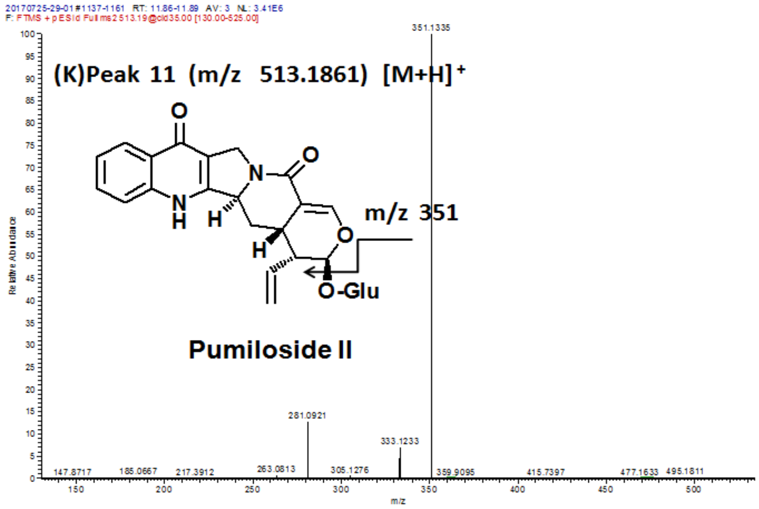
(K)Peak 11 (m/z 513.1861) [M+H]+
O
N
O
N
H
H
m/z 351
O
H
O-Glu
Pumiloside II
351.1335
281.0921
333.1233
Relative Abundance
m/z

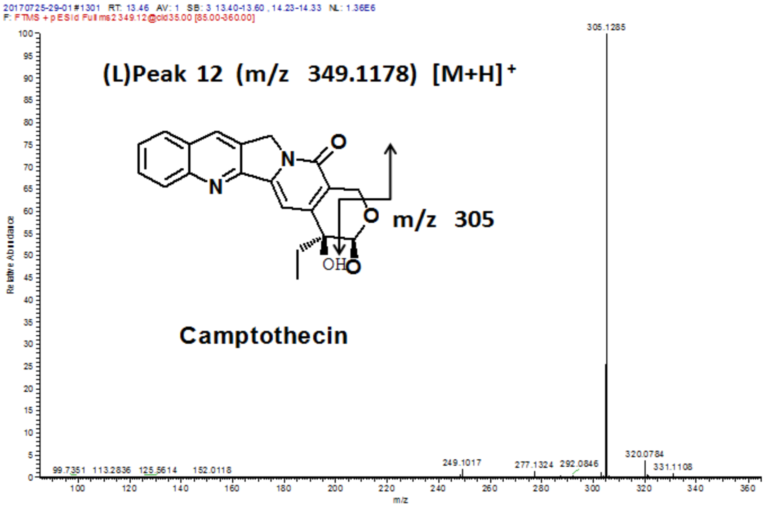
(L)Peak 12 (m/z 349.1178) [M+H]+
N
O
N
O
m/z 305
OH
O
Camptothecin
305.1285
320.0784
331.1108
249.1017
Relative Abundance
m/z

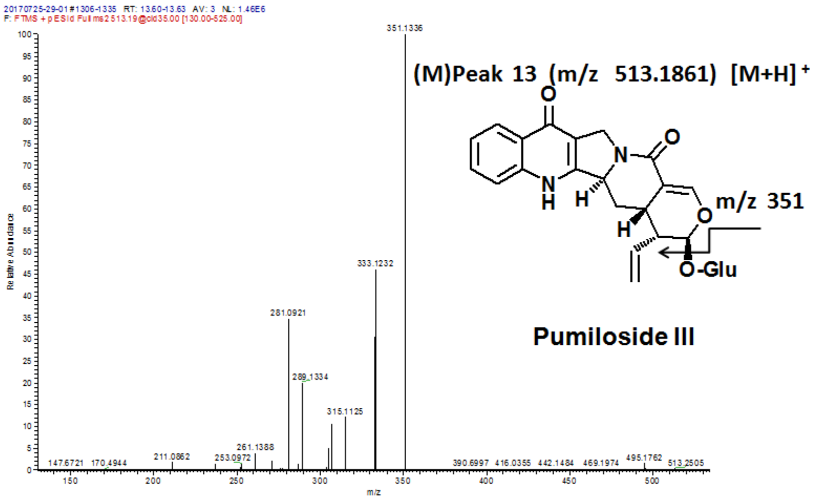

(M)Peak 13 (m/z 513.1861) [M+H]+
m/z 351
O-Glu
Pumiloside III
351.1336
333.1232
281.0921
289.1334
315.1125
261.1388
Relative Abundance
m/z

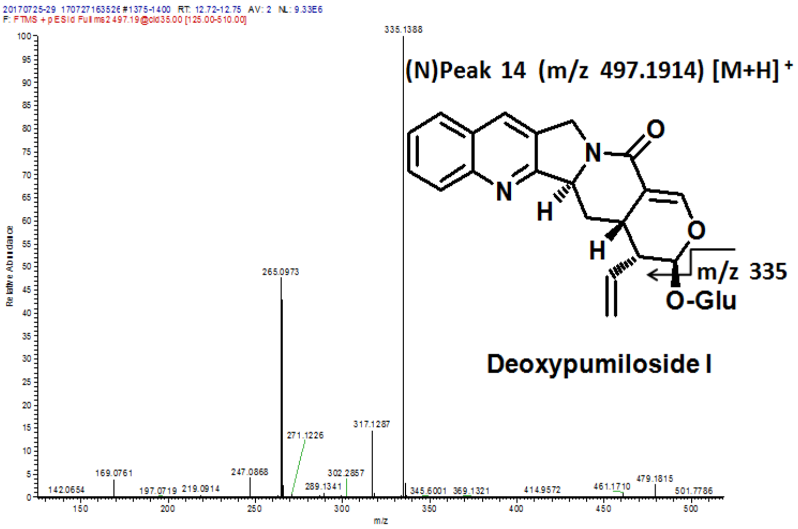

(N)Peak 14 (m/z 497.1914) [M+H]+
m/z 335
O-Glu
Deoxypumiloside I
335.1388
265.0973
317.1287
271.1226
247.0868
169.0761
Relative Abundance
m/z

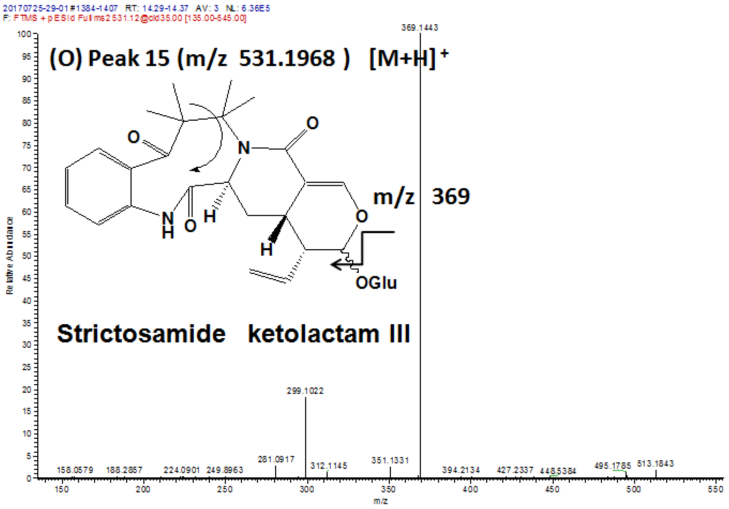

(O) Peak 15 (m/z 531.1968) [M+H]+
m/z 369
OGlu
Strictosamide ketolactam III
369.1443
299.1022
351.1331
281.0917
513.1843
Relative Abundance
m/z

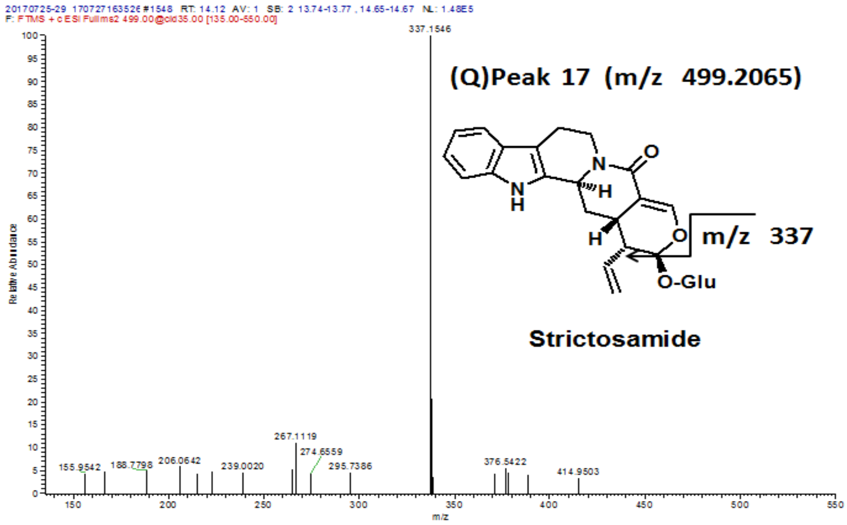

20170725-29 170727163526 #1548 RT: 14.12 AV: 1 SB: 2 13.74-13.77 , 14.65-14.67 NL: 1.48E5
F: FTMS + c ESI Full ms2 499.00@cid35.00 [135.00-550.00]
Relative Abundance
m/z
337.1546
267.1119
274.6559
295.7386
155.9542
188.7798
206.0642
239.0020
376.5422
414.9503
(Q)Peak 17 (m/z 499.2065)
m/z 337
O-Glu
Strictosamide

Fig. S3

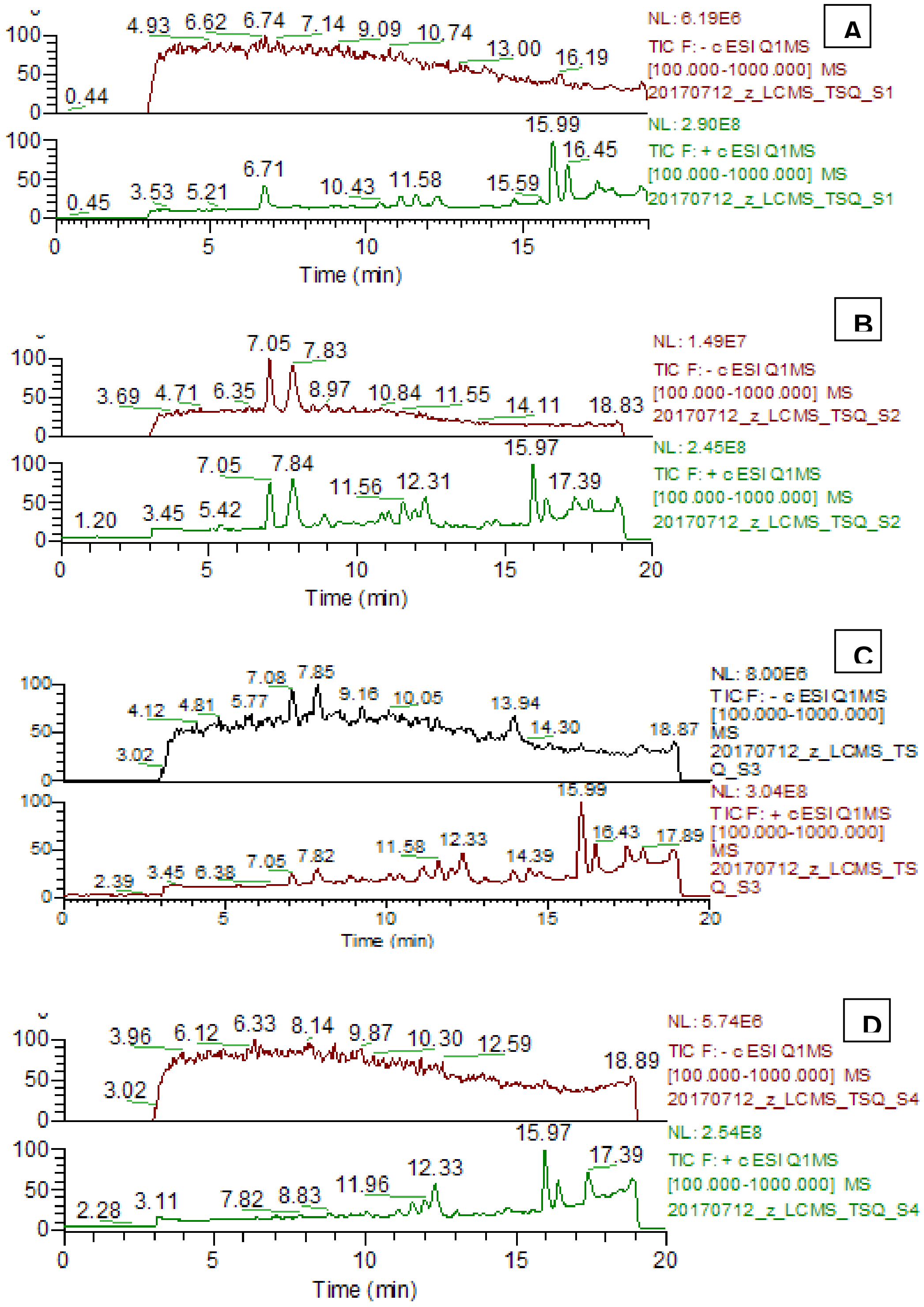
A
NL: 6.19E6
TIC F: - c ESI Q1MS
[100.000-1000.000] MS
20170712_z_LCMS_TSQ_S1
NL: 2.90E8
TIC F: + c ESI Q1MS
[100.000-1000.000] MS
20170712_z_LCMS_TSQ_S1
4.93 6.62 6.74 7.14 9.09 10.74 13.00 16.19 0.44
15.99 16.45 6.71 10.43 11.58 15.59 0.45 3.53 5.21
100 50 0
0 5 10 15
Time (min)
B
NL: 1.49E7
TIC F: - c ESI Q1MS
[100.000-1000.000] MS
20170712_z_LCMS_TSQ_S2
NL: 2.45E8
TIC F: + c ESI Q1MS
[100.000-1000.000] MS
20170712_z_LCMS_TSQ_S2
7.05 7.83 3.69 4.71 6.35 8.97 10.84 11.55 14.11 18.83
15.97 7.05 7.84 11.56 12.31 17.39 1.20 3.45 5.42
100 50 0
0 5 10 15 20
Time (min)
C
NL: 8.00E6
TIC F: - c ESI Q1MS
[100.000-1000.000]
MS
20170712_z_LCMS_TS
Q_S3
NL: 3.04E8
TIC F: + c ESI Q1MS
[100.000-1000.000]
MS
20170712_z_LCMS_TS
Q_S3
7.08 7.85 4.12 4.81 5.77 9.16 10.05 13.94 14.30 18.87 3.02
15.99 16.43 17.89 7.05 7.82 11.58 12.33 14.39 2.39 3.45 6.38
100 50 0
0 5 10 15 20
Time (min)
D
NL: 5.74E6
TIC F: - c ESI Q1MS
[100.000-1000.000] MS
20170712_z_LCMS_TSQ_S4
NL: 2.54E8
TIC F: + c ESI Q1MS
[100.000-1000.000] MS
20170712_z_LCMS_TSQ_S4
3.96 6.12 6.33 8.14 9.87 10.30 12.59 18.89 3.02
15.97 17.39 12.33 11.96 2.28 3.11 7.82 8.83
100 50 0
0 5 10 15 20
Time (min)
